# Influence of Simulated Deep Brain Stimulation on the Expression of Inflammatory Mediators by Human Central Nervous System Cells In Vitro

**DOI:** 10.1007/s12017-021-08674-y

**Published:** 2021-07-03

**Authors:** Carolin Kubelt, Henri Molkewehrum, Ralph Lucius, Michael Synowitz, Janka Held-Feindt, Ann-Kristin Helmers

**Affiliations:** 1 Department of Neurosurgery, University Medical Center Schleswig-Holstein UKSH, Campus Kiel, Arnold-Heller-Str. 3, House D, 24105 Kiel, Germany; 2 Department of Anatomy, University of Kiel, 24118 Kiel, Germany

**Keywords:** DBS, In-vitro model, Inflammation, Chemokines, Cytokines

## Abstract

Deep brain stimulation (DBS) seems to modulate inflammatory processes. Whether this modulation leads to an induction or suppression of inflammatory mediators is still controversially discussed. Most studies of the influence of electrical stimulation on inflammation were conducted in rodent models with direct current stimulation and/or long impulses, both of which differ from the pattern in DBS. This makes comparisons with the clinical condition difficult. We established an in-vitro model that simulated clinical stimulation patterns to investigate the influence of electrical stimulation on proliferation and survival of human astroglial cells, microglia, and differentiated neurons. We also examined its influence on the expression of the inflammatory mediators C-X-C motif chemokine (CXCL)12, CXCL16, CC-chemokin-ligand-2 (CCL)2, CCL20, and interleukin (IL)-1β and IL-6 by these cells using quantitative polymerase chain reaction. In addition, protein expression was assessed by immunofluorescence double staining. In our model, electrical stimulation did not affect proliferation or survival of the examined cell lines. There was a significant upregulation of CXCL12 in the astrocyte cell line SVGA, and of IL-1β in differentiated SH-SY5Y neuronal cells at both messenger RNA and protein levels. Our model allowed a valid examination of chemokines and cytokines associated with inflammation in human brain cells. With it, we detected the induction of inflammatory mediators by electrical stimulation in astrocytes and neurons.

## Introduction

Deep brain stimulation (DBS) is an established therapy for movement disorders (Deuschl & Agid, 2013; Huss et al., 2015; Janssen et al., 2014) and is under investigation for pain and for psychiatric indications such as depression and obsessive–compulsive disorder (Fenoy et al., 2018; Frizon et al., 2020; Huys et al., 2019). The complex molecular effects of DBS are still not fully understood and seem to be far beyond a simple initially considered mimicking of a lesion. There is increasing evidence that electrical stimulation modulates inflammatory processes. However, whether DBS leads to an unwanted induction or beneficial suppression of immune response is still controversially discussed.

It is currently assumed that the phenomenon of a non-hemorrhagic, non-infectious delayed-onset edema surrounding deep brain-stimulated areas is not only caused by the implantation of the electrode itself but also by local inflammatory processes in the context of electrical stimulation (Cuba et al., 2016; Saitoh et al., 2019). One major worry in the field of a local immune response is the development of a glial scar around the DBS electrode, which might impede or even interrupt the electrical output (McConnell et al., 2009; Pflüger et al., 2019). Scientists are intensively working on the further development of the implanted material in order to reduce an immune reaction due to the implant itself (Gulino et al., 2019). However, the impact of the electrical stimulation on local inflammatory processes still remains. A promotion of inflammation due to DBS not only seems to be a local phenomenon. DBS in specific brain regions was also shown to induce a systemic immune response. Calleja-Castillo et al. found increased levels of circulating tumor necrosis factor (TNF)-α, interleukin (IL)-1β, IL-6, and interferon-γ, all cytokines known to be involved in inflammation, after 21 days of DBS of the hypothalamic nucleus in a rat model (Calleja-Castillo et al., 2013). Moreover, they observed decreased serum concentrations of the anti-inflammatory steroid hormone corticosterone in the examined animals (Calleja-Castillo et al., 2013).

In contrast to the literature previously cited, other studies postulated a neuroprotective effect and observed neural circuits to be modulated by electrical stimulation of distinct brain regions leading to a reduction of inflammatory mediators, for example, in ischemic brain regions (Baba et al., 2009; Schuhmann et al., 2019). Electrical stimulation was even shown to lead to smaller volumes of stroke areas due to a reduction of inducible nitric oxide synthase expression (Galea et al., 1998). Moreover, Chen et al. showed that electrical stimulation of anterior thalamic nuclei in the field of epilepsy downregulated inflammatory processes in the hippocampus, leading to a reduction of neuronal loss and neurogenesis in a rat model (Chen et al., 2017). Similar results were obtained in a Parkinson disease (PD) rat model, in which subthalamic nucleus-DBS-suppressed neuroinflammation and led to an increased survival of dopaminergic neurons in the substantia nigra (Chen et al., 2020). In addition, Dandekar et al. showed a significant downregulation of the inflammatory mediators IL-5 and IL-18 in the hippocampus and of IL-6 in the nucleus accumbens after seven days of DBS of the medial forebrain bundle in a depression model (Dandekar et al., 2019). Furthermore, they found higher levels of brain-derived neurotrophic factor (BDNF) in plasma, cerebrospinal fluid (CSF), and hippocampus following DBS. BDNF is one of the major mediators of neuroplasticity and has been shown to be downregulated by proinflammatory cytokines (Calabrese et al., 2014).

Apart from neural circuits triggered by the electrical stimulation of distinct brain regions, it is of main interest, which basic immunological effects of electrical current have on the different cell types of the central nervous system. Campos et al. investigated the effect of high-frequency stimulation in cultured astrocytes and observed an activation of astrocytes and a prevention of TNF-α-induced increase of monocyte chemoattractant protein-1 (MCP-1) and NF-κB activation in vitro (Campos et al., 2020). In further research focusing on the general influence of electrical current on the different cell types of the central nervous system, Pelletier et al. observed longer somata of neurons and astroglia due to stimulation in vitro, and Li et al. found an upregulation of the mitogen-activated protein kinase pathway in oligodendroglia precursor cells leading to migration towards the anode when an electric field of 200 mV/mm was applied (Li et al., 2015; Pelletier et al., 2014). A further in-vivo study of Keuters et al. also showed a greater migration of neuronal progenitor cells after being exposed to transcranial or direct stimulation (Keuters et al., 2015).

Anyway, little is known about the effects of electrical stimulation on inflammatory processes in various human brain cell types. In addition, previous in-vitro studies in human brain cells have mostly used direct current or impulses longer than normally employed in routine clinical practice.

In order to investigate the influence of DBS on proinflammatory cytokines and chemokines of cells of the human central nervous system, we established an in-vitro model that closely simulates the current standard stimulation conditions for patients using an indirect application of the current. Understanding the underlying mechanism of the interplay between electrical stimulation and inflammation is imperative to hinder negative side effects and use its beneficial impact.

## Materials and Methods

### Cell Lines

The human fetal astrocyte cell line SVGA, the human microglial cell line HMC3, and the human neuroblastoma cell line SH-SY5Y were used for our investigations. The SVGA cells were kindly provided by the group of Christine Hanssen Rinaldo, University Hospital of North Norway (Henriksen et al., 2014) with the permission of Altwood (Schweighardt et al., 2001). The HMC3 and SH-SY5Y cells were purchased from the American Type Culture Collection (ATCC, Manassas, Virginia, USA). SVGA and HMC3 cells were cultured in Dulbecco’s modified Eagle’s medium (DMEM; Life Technologies, Carlsbad, CA, USA). SH-SY5Y cells were cultured in a half and half mixture of F12 (ATCC) and Eagle’s minimum essential medium (EMEM). The corresponding media were supplemented with 10% fetal bovine serum (FBS; PAN-Biotech GmbH, Aidenbach, Germany), 1% penicillin–streptomycin (10,000 U/ml; Thermo Fisher Scientific, Waltham, MA, USA), and 2 mM of additional l-glutamine (Thermo Fisher Scientific). Purity of the cells was ascertained by immunostaining with cell type-specific markers and by the absence mycoplasma contamination. Cell line identity was verified by short tandem repeat profiling as previously described (Adamski et al., 2017).

### Differentiation of SH-SY5Y to Dopaminergic-Like Neurons

Before using the SH-SY5Y cells, they were first triggered to differentiate into dopaminergic-like neurons. For this, the SH-SY5Y cells were seeded in six-well plates with a density of 200,000 cells per well. After 48 h of cultivation in the incubator (37 °C, 5% CO_2_), the undifferentiated cells were rinsed with phosphate-buffered saline (PBS), the medium was aspirated and replaced by an EMEM/F12 stimulation medium containing 10% FBS and 10 μM all-trans retinoic acid (RA, Merck SIGMA-ALDRICH, Germany). After a further 48 h of cultivation, the medium was gently aspirated, new medium containing 5% FBS and 10 μM RA was added, and the cells were returned to the incubator. After a further 48 h, the medium was gently aspirated off once again, and the cells were rinsed in PBS. PBS was aspirated and new medium containing 1% FBS and 10 μM RA was added. After a further 72 h of incubation, this medium was replaced with FBS-free EMEM/F12 medium, and the cells were ready for use.

### In-vitro Model of Simulated DBS

**Fig. 1:**
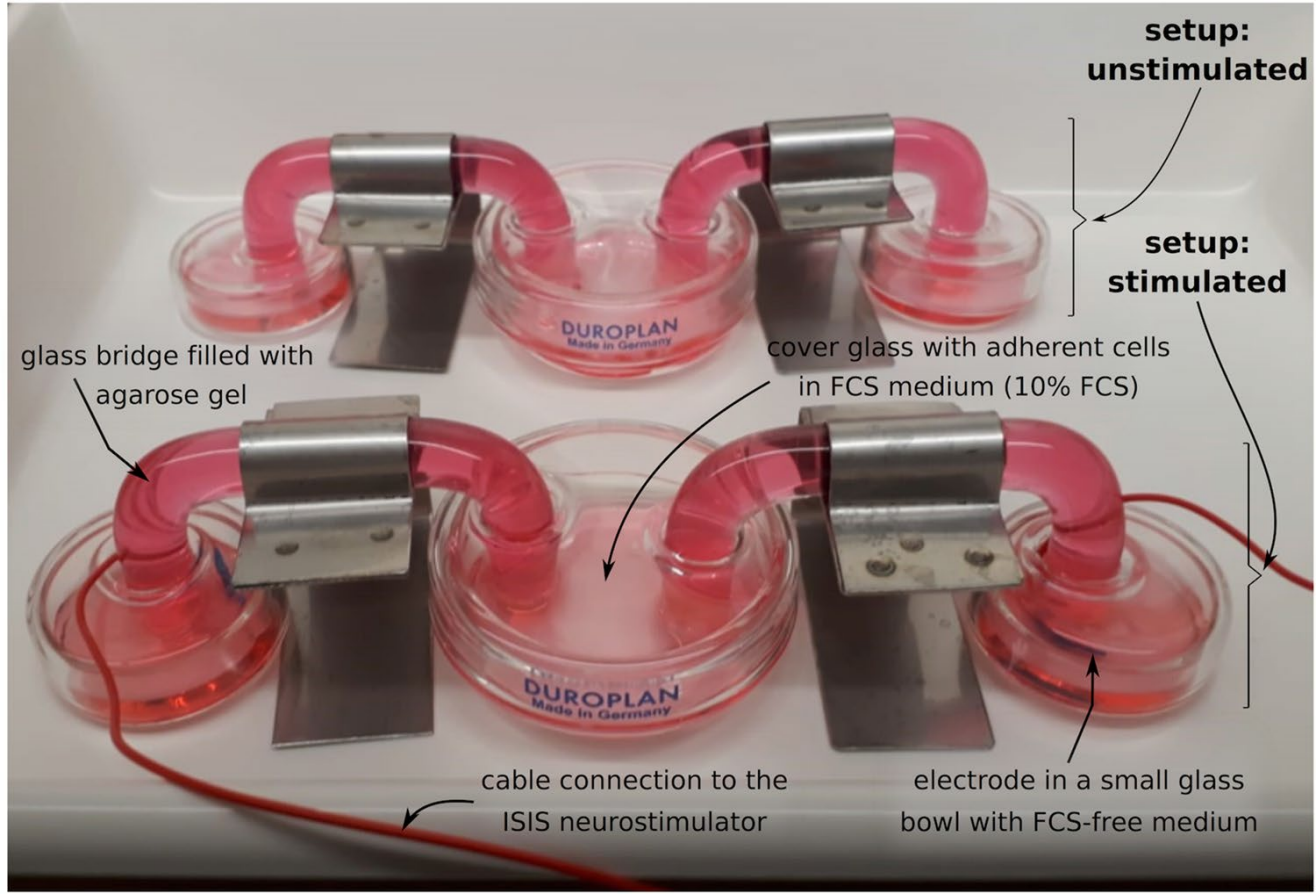
Experimental setup of the in-vitro model of DBS. Adherent cells of different cell lines were electrically stimulated indirectly via glass bridges filled with agarose gel. The unstimulated control was performed equally except omitting the electrodes.

Adherent SVGA and HMC3 cells, and differentiated SH-SY5Y cells were seeded onto coverslips (200,000 per coverslip) 24 h before electrical stimulation. The coverslips were then transferred onto glass bowls containing 5 ml DMEM supplemented with 10% FBS. During the experiment, the cells were kept in an incubator at 37 °C and 5% CO_2_. The tips of the neurostimulator electrodes were submerged in glass bowls filled with 2 ml DMEM without FBS (Fig. 1). To prepare the glass bridges, which connected the different glass bowls, 0.5% agarose powder (SIGMA-ALDRICH, St. Louis, Missouri, USA) was dissolved in DMEM (Life Technologies) without FBS and briefly brought to a boil. The glass tubes were then filled with the liquid, which was allowed to solidify at room temperature. All glass bowls were covered to prevent evaporation with a resulting shift in ion concentrations in the cultivation medium. The Inomed system for intraoperative stimulation (ISIS®), a multifunctional voltage stimulator conceived specifically for intraoperative neurostimulation and operated with the ISIS® stimulation software (Inomed Medizintechnik GmbH, Emmendingen, Germany) was used. To achieve constant test conditions, the current between the glass bridges was measured with an oscilloscope (LIUMY Multimeter, LM2001, Shenzhen Yisi Technology Co., ShenZhen, China).

### Cell Count

To perform the cell count, the medium was aspirated and the cells were rinsed with PBS. After aspirating the PBS, 600 μl of trypsin/EDTA (1%, 10×, T4174, SIGMA-ALDICH) was added and the samples were incubated at 37 °C for 5 min to detach the cells from the surface. The detached cells were resuspended in 2 ml of DMEM, resp. EMEM/F12 medium and transferred to a centrifuge tube. After spinning them down for five min at 0.3 RCF and aspirating the supernatant, the cell pellet was resuspended in 2 ml DMEM or EMEM/F12. Ten μl of the cell-containing medium was mixed with 10 μl of trypan blue (0.4%, MERCK, Darmstadt). Ten μl of this suspension was pipetted into a Neubauer counting chamber (ASSISTENT-Zählkammer, Glaswarenfabrik Karl Hecht, Sonndheim vor der Rhön, Germany). Apoptotic or necrotic cells stained by the trypan blue were excluded from the count. The unstained cells in four quadrants were counted and the total cell number was calculated. Phase contrast photomicrographs were taken with Axiovert 40 CFL, Carl Zeiss, Oberkochen, Germany.

### Quantitative Reverse Transcription-Polymerase Chain Reaction (qRT-PCR)

RNAs of the stimulated and unstimulated probes of each cell line were isolated with the ARCTURUS® PicoPure® RNA Isolation Kit (Applied Biosystems, Waltham, MA, USA) according to the manufacturer’s instructions at 0 h and 24 h of stimulation. DNase digestion (Promega, Madison, WI, USA), cDNA synthesis using RevertAid™ H Minus Reverse Transcriptase (Thermo Fisher Scientific), and qRT-PCR using TaqMan primer probes (Applied Biosystems) were performed as described earlier (Hattermann et al., 2010). The primers used were CXCL12 (Hs00171022_m1), CXCL16 (Hs00222859_m1), CCL2 (Hs_00234140_m1), CCL20 (Hs_00171125_m1), IL-1β (Hs_01555410_m1), IL-6 (Hs_00985639_m1), and glycerinaldehyde 3-phosphate dehydrogenase (GAPDH) (Hs-99999905_m1). Fluorescent data were converted into cycle threshold (*C*_T_) measurements and Δ*C*_T_ values of each sample were calculated as *C*_T gene of interest_ − *C*_T GAPDH_. The induced gene expression is displayed as n-fold expression changes $= 2^{{ - \left( {\Delta {\text{CT}}\;{\text{stimulated}}\, - \,\Delta {\text{CT}}\;{\text{unstimulated}}} \right)}}$.

### Immunocytochemistry

Immunocytochemistry was performed with differentiated and undifferentiated SH-SY5Y cells as well as with stimulated and unstimulated SVGA cells. After washing the cells three times in 2 ml PBS, they were fixed in 2 ml ice-cold methanol–acetone (1:1) for 10 min. Afterwards, the cells were washed again three times in PBS, and unspecific binding was blocked by incubation in 100 µl PBS, 0.1% bovine serum albumin (BSA Fraction V; Serva Electrophoresis GmbH), and 0.2% glycine for 60 min. The cells were then incubated with the primary antibody overnight at 4 °C in a humid chamber. The primary antibodies were anti-NF200 (1:1,000, MAB5266, mouse IgG; SIGMA-ALDRICH), anti-dopamine (1:350, sc-51871, mouse IgG; Santa Cruz Biotechnology, Dallas, Texas, USA), anti-CXCL12 (1:100, sc-28876, rabbit IgG; Santa Cruz Biotechnology) and anti-IL-1β (1:100, sc-7884, rabbit IgG; Santa Cruz Biotechnology). After washing the cells three times in 2 ml PBS, they were incubated with the secondary antibody Alexa Fluor 488 (1:1,000, A21201, donkey-anti-rabbit IgG; Invitrogen Molecular Probes, Walham, Massachusetts, USA) for 1 h at 37 °C protected from light in a humid chamber. After washing the cells again in PBS, the nuclei were counterstained with 4′,6-diamidino-2-phenylindole (DAPI; 1:30,000, SIGMA-ALDRICH) by incubation for 30 min at room temperature. Afterwards, the cells were washed three times in 2 ml PBS and then once in distillated water. The primary antibodies were omitted for negative controls. For secondary antibody controls, mouse IgG (MAB002, R&D Systems, Inc., Minneapolis, USA) control antibodies were used instead of the primary antibodies at the same concentrations as the replaced primary antibodies. Sections were embedded with Immumount (Thermo Fisher Scientific), and fluorescence signals were analyzed using an AxioObserver.Z1 microscope (Zeiss).

### TUNEL (Terminal Deoxynucleotidyl Transferase-Mediated d-UTP Nick End Labeling) Assay

Apoptosis due to electrical stimulation was detected by TUNEL assays using CF® dye TUNEL Assay Apoptosis Detection Kit (Biotium, Inc., Fremont, CA) performed after 24 h of stimulation. This assay relies on detectioning the DNA strand breaks (DSB) that occur during apoptosis by labeling them with fluorochromes. Apoptotic cells can be hence identified and quantified by fluorescence microscopy. In this procedure, the 3′OH-termini of the DSBs serve as primers and become labeled with fluorochrome-tagged deoxyuridine triphosphate (d-UTP) in a reaction catalyzed by exogenous terminal deoxynucleotidyl transferase (TdT; Biotium).

The cells were first washed twice in PBS and briefly fixed in 4% paraformaldehyde (Merck) and PBS for 30 min at 4 °C. After washing them twice in PBS again, the cells were permeabilized in PBS containing 0.2% Triton X-100 (Merck) for 30 min at room temperature. After washing the cells twice again in PBS, they were incubated with 100 µl TUNEL equilibration buffer (Biotium) for 5 min. After aspirating the equilibration buffer in a dark room, 50 µl TUNEL Reaction Buffer (Biotium) was supplemented with 1 µl TdT-enzyme (Biotium) and added to each probe. After cell staining (see “Immunocytochemistry” section), the cells were washed three times for 5 min in PBS containing 0.1% Triton X-100 and 5 mg/ml BSA. As a positive control, cells were incubated with the cytostatic drug camptothecin (stock solution: 10 µg/µl; 1 µl applied, C9911, Merck SIGMA-ALDRICH) for 24 h before performing the TUNEL assay. This agent inhibits the enzyme topoisomerase 1, which counteracts excessive twisting of the DNA during replication. A malfunction of this enzyme, thus, leads to an excessive twisting of the DNA causing strand breaks and the initiation of apoptosis. Cells were counterstained with DAPI, embedded with Immumount® (Thermo Fisher Scientific), and fluorescence signals were analyzed using an AxioObserver.Z1 microscope (Zeiss). The samples were stored at 4 °C protected from light.

### Statistical Analysis

Results are expressed by means ± SD. Statistical analysis was performed using either two-way analysis of variance (ANOVA) with Bonferroni posttests or paired two-tailed Student’s t test that particular test was used, which is indicated in the figure legends, respectively. Significance levels were *p* < 0.05 (indicated by *), *p* < 0.01 (indicated by **), and *p* < 0.001 (indicated by ***). Data management and statistical analysis were performed using the Graphpad Prism 9.0.0 ® software (GraphPad Software, San Diego, CA, USA).

## Results

### Establishment of the Simulated Deep Brain Stimulation In-Vitro Model

In order to examine the effects of DBS on various human cell types (astroglial cells, microglia, neurons), one requires an experimental in-vitro setup that reproduces the clinical conditions as nearly as possible. Thus, first of all, we established an in-vitro model of DBS with a distinct voltage ratio using short impulse widths (60 µs) and high frequency (130 Hz), as used in clinical care in patients (Fig. 1). Stimulation was carried out for up to 24 h.

The voltage actually applied to the cells and generated by the ISIS® neurostimulator was 2 mV. The stimulation was carried out indirectly by placing the ends of the electrodes in small glass bowls, which were connected to the coverslips, placed in glass bowls and containing the cells to be examined, via glass bridges filled with agarose gel. This setup prevented an unwanted anion deposition at the ends of the electrodes, which could otherwise falsify the results of the experiment. By placing the coverslips with the adherent cells accurately central in the electron flow, an exact electrical stimulation is applied. The experimental setup of the unstimulated control was equal, except of the omitted electrodes.

**Fig. 2:**
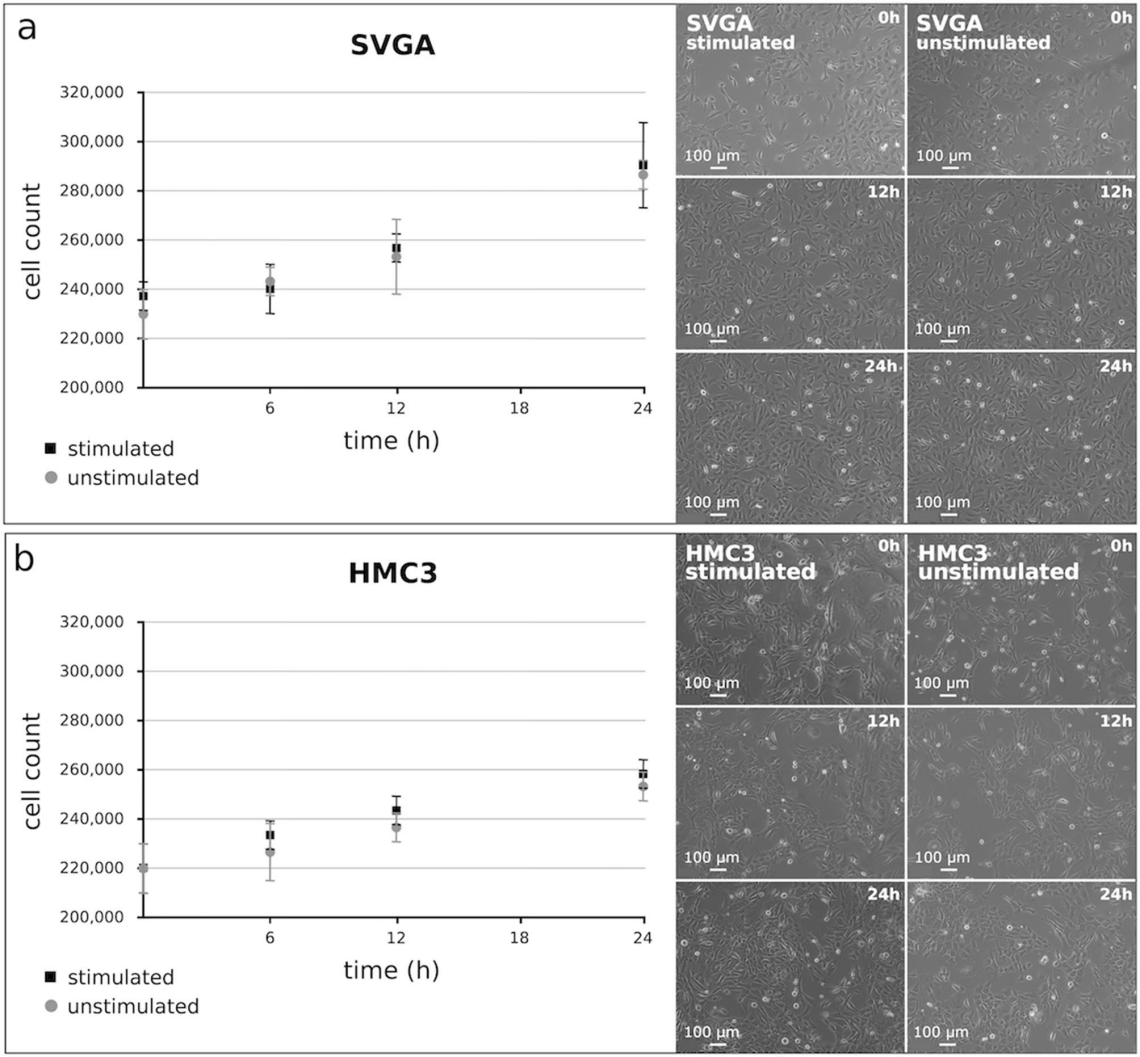
Cell count of electrically stimulated (2 mV) and unstimulated **A** SVGA and **B** HMC3 cells at different time points during the stimulation (0 h, 6 h, 12 h, 24 h). The cell counts (*n* = 3) are shown on the left (mean ± SD), whereas the right side shows the photomicrographs of the cells at the corresponding time points. Magnification ×100; bar = 100 μm. Using two-way ANOVA with Bonferroni posttests, no statistically differences were found concerning the growth behavior of the stimulated and unstimulated cells in the two cell lines, respectively

To test whether electrical stimulation influences the proliferation of the different human cell types, a cell count was performed at different time points during stimulation (0 h, 12 h, 24 h) and photomicrographs were taken. Comparing stimulated and unstimulated cells, neither statistically significant nor optical differences were found concerning the growth behavior of the SVGA (astroglial cells) or the HMC3 (microglia) cells. Both cells lines exhibited a constant and nearly linear growth. After 24 h of stimulation, the cell count increased by an average of 25% in SVGA cells and 16% in HCM3 cells (Fig. 2).

**Fig. 3:**
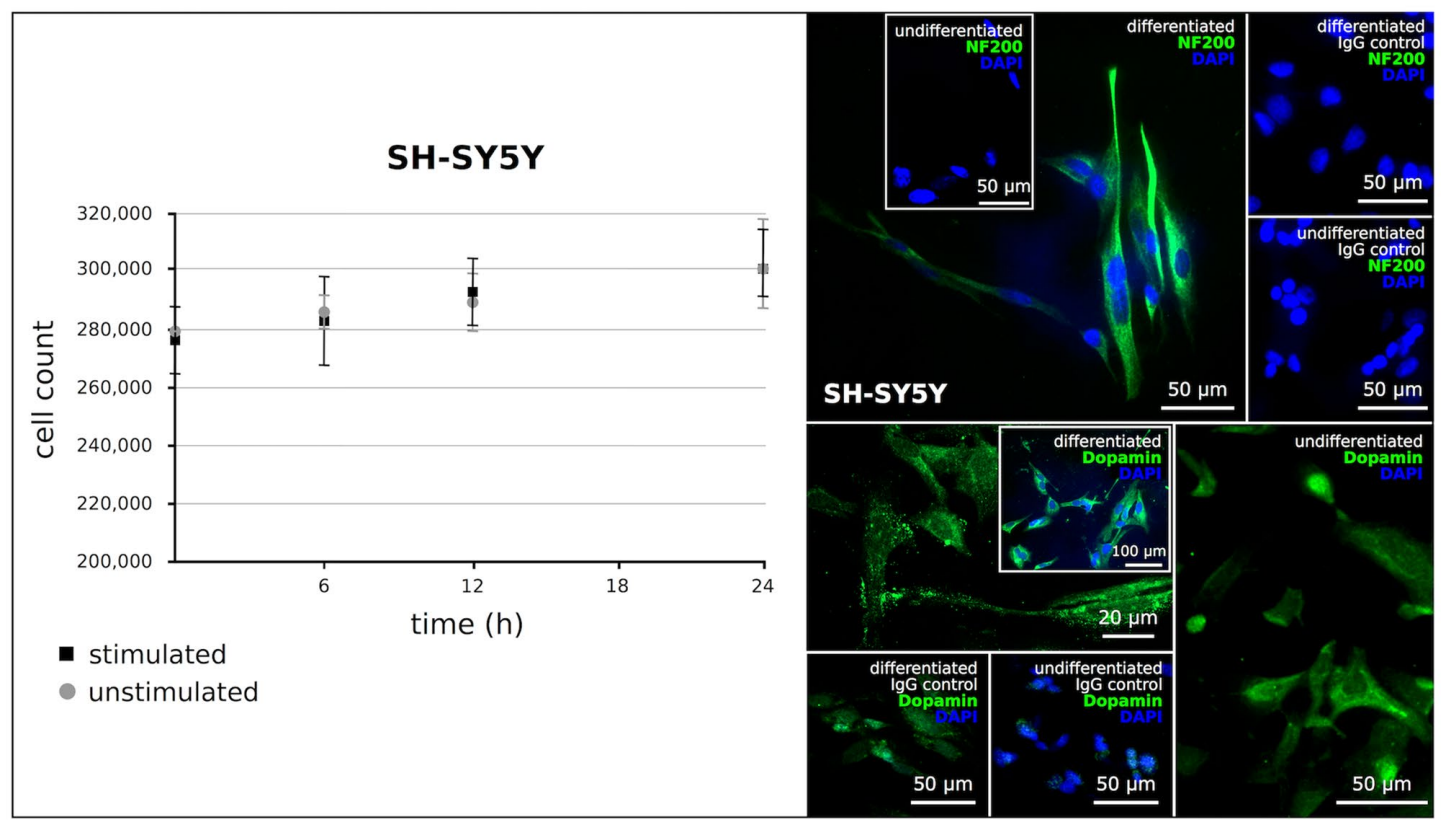
Left: Cell counts (*n* = 3) of electrically stimulated and unstimulated differentiated SH-SY5Y cells at various time points during stimulation (mean ± SD); there was no statistically difference of the growth behaviors of the stimulated and unstimulated cells as determined by two-way ANOVA with Bonferroni posttests, and the growth behaviors were even nearly identical. Right: Immunofluorescence staining to document the differentiation of the SH-SY5Y cells to dopaminergic-like neurons using the neuronal marker NF (neurofilament) 200 and dopamine; both markers are induced in the differentiated cells; Magnification ×200; bar = 100 μm, 50 μm or 20 μm

Furthermore, the influence of electrical stimulation on the proliferation of differentiated, dopaminergic-like human neurons was examined by cell counting. For differentiation, SH-SY5Y (neuroblastoma) cells were exposed to retinoic acid and the success of the treatment was proven via immunofluorescence staining with the neuronal marker NF (neurofilament) 200 and dopamine. In relation to undifferentiated controls, both markers were clearly induced in the differentiated cells. A count of the electrically stimulated and differentiated SH-SY5Y cells compared with the unstimulated differentiated control cells did not show any significant difference in growth behavior. In general, the SH-SY5Y cells only exhibited a slight linear growth. Since these cells are differentiated, postmitotic neurons, proliferation was not expected. The observed low growth seems to be due to a small number of cells that had remained undifferentiated. The cell counts, as well as the visualization of the degree of differentiation of the SH-SY5Y cells to dopaminergic-like neurons by immunofluorescence staining are presented in Fig. 3.

**Fig. 4:**
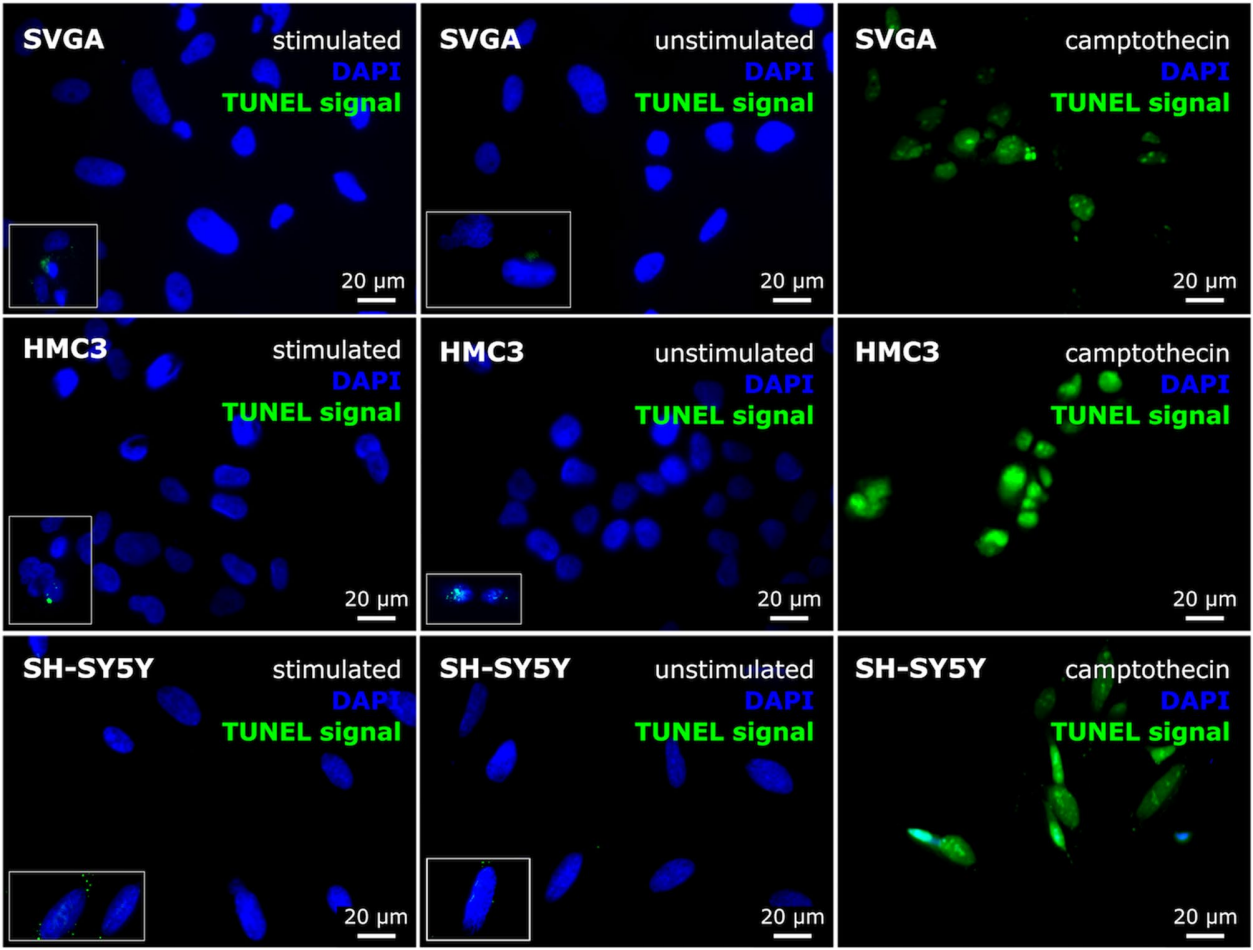
Results of the TUNEL (terminal deoxynucleotidyl transferase d-UTP nick end labeling) assay comparing the apoptotic behavior of electrically stimulated and unstimulated SVGA, HCM3, and SH-SY5Y cells. Stimulation with the cytostatic drug camptothecin served as a positive control. The solely qualitative evaluation revealed no differences for any cell line between the staining of the nuclei (DAPI) of stimulated and unstimulated cells. In opposite to the positive controls, TUNEL signal indicating apoptosis was only seen in rare cases of stimulated and unstimulated cells. Accordingly, the nuclei of the positive control cells exhibited only weak or barely visible staining. Magnification ×400; bar = 20 μm

In a next step, we examined the effects of electrical stimulation on cell survival. For this, the SVGA, HMC3 and the differentiated SH-SY5Y cells were electrically stimulated for 24 h. In addition to an unstimulated control, stimulation with the cytostatic drug camptothecin was performed. This agent initiates apoptosis, and the cells, therefore, served as a positive control. A TUNEL (terminal deoxynucleotidyl transferase d-UTP nick end labeling) assay was used to evaluate the effects of the stimulation on cell survival. This method tags apoptotic DNA fragments with a fluorochrome. No differences were observed in the solely qualitative evaluation of the staining of the cell nuclei between stimulated and unstimulated cells in any examined cell lines. In addition, nearly no DNA fragmentation occurred in these cells indicated by only rare green fluorescence signal, while all positive control cells exhibited a strong green fluorescence signal indicating apoptosis. Furthermore, very weak, or even no longer detectable, nuclear staining was found in the camptothecin-stimulated probes as a sign of cell death. Figure 4 shows the results of the TUNEL assay.

The previous observation that electrical stimulation did not affect cell proliferation or survival in our experimental setup in any cell line established a basis for the following analysis of the regulation of inflammatory mediators under electrical stimulation.

### The Influence of the Electrical Stimulation on Inflammatory Mediators

First, in order to determine the effects of the electrical stimulation on particular chemokines and cytokines involved in inflammation, qRT-PCR was carried out.

**Fig. 5:**
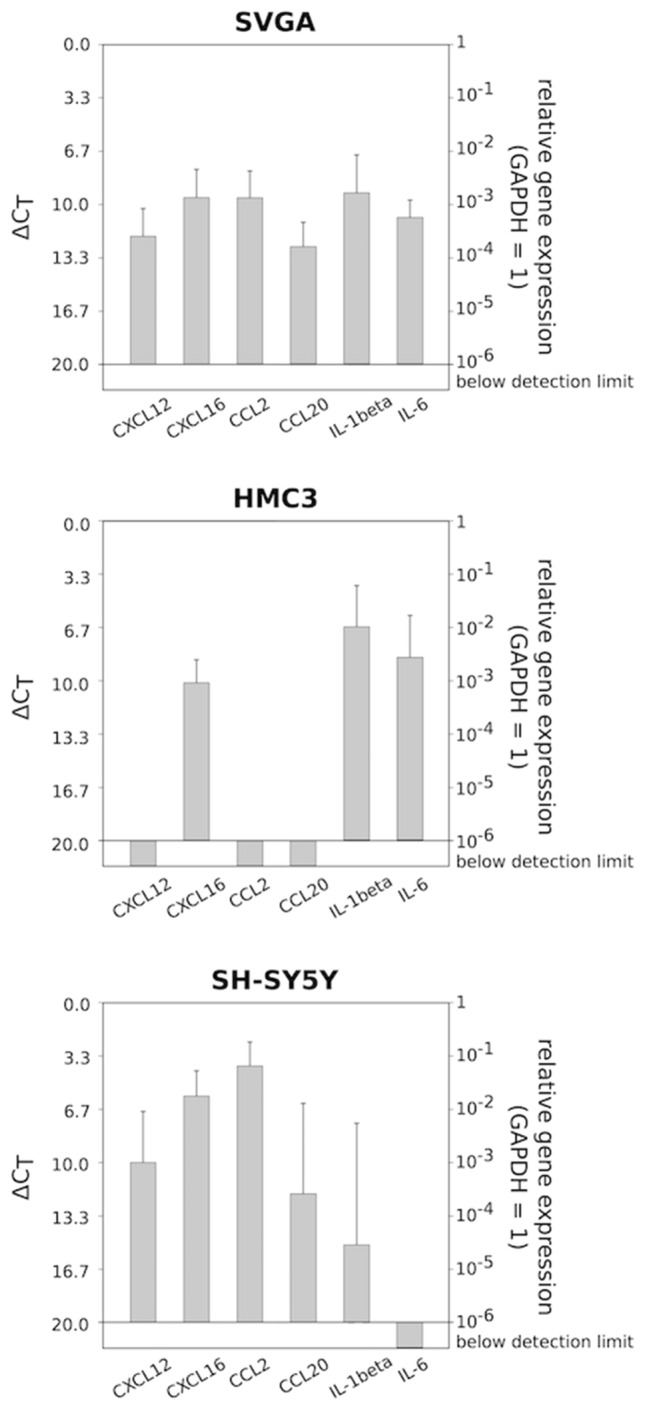
Basal gene expression of CXCL12, CXCL16, CCL2, CCL20, IL-1β, and IL-6 in SVGA (*n* = 8), HMC3 (*n* = 9), and SH-SY5Y (*n* = 9) cells determined by qRT-PCR (logarithmic scale, a Δ*C*_T_ = 3.33 increase corresponds to a tenfold decrease in gene expression). While SVGA cells expressed all examined mediators, HMC3 cells did not express CXCL12, CCL2, or CCL20, and SH-SY5Y cells did not express IL-6

First of all, the basal gene expression of CXCL12, CXCL16, CCL2, CCL20, IL-1β, and IL-6 was determined in SVGA, HMC3, and SH-SY5Y cells using GAPDH as internal standard (Fig. 5). A Δ*C*_T_ value of 3.33 corresponds to a one order of magnitude lower gene expression. While all chemokines and cytokines were clearly detectable in SVGA cells, no expression of CXCL12, CCL2, or CCL20 was found in HMC3 cells, and SH-SY5Y cells did not express IL-6. Of all examined mediators, CCL2 (average Δ*C*_T_: 3.89; SD: 1.51) showed the highest gene expression, followed by CXCL16 (average Δ*C*_T_: 5.76; SD: 1.59) in SH-SY5Y cells, and IL-1β (average Δ*C*_T_: 6.57; SD: 2.58) in HMC3 cells. IL-6 (average Δ*C*_T_: 10.72; SD: 1.05), CXCL16 (average Δ*C*_T_: 9.47; SD: 1.71), CCL2 (average Δ*C*_T_: 9.94; SD: 1.63), and IL-1β (average Δ*C*_T_: 9.19; SD: 2.38) in SVGA cells, CXCL16 (average Δ*C*_T_: 10.03; SD: 1.39) and IL-6 (average Δ*C*_T_: 8.47; SD: 1.92) in HMC3 cells, and CXCL12 (average Δ*C*_T_: 9.87; SD: 3.17) in SH-SY5Y cells revealed moderate gene expression levels. The lowest gene expression level was found for IL-1β in SH-SY5Y cells (average Δ*C*_T_: 14.96; SD: 7.52) followed by CCL20 (average Δ*C*_T_: 12.52; SD: 1.49) and CXCL12 (average Δ*C*_T_: 11.85; SD: 1.69) in SVGA cells, and CCL20 (average Δ*C*_T_: 11.81; SD: 5.62) in SH-SY5Y cells. Overall, most of the investigated cytokines and chemokines were clearly detectable in the investigated cell lines, but the degree of gene expression differed between the individual cell lines.

**Fig. 6:**
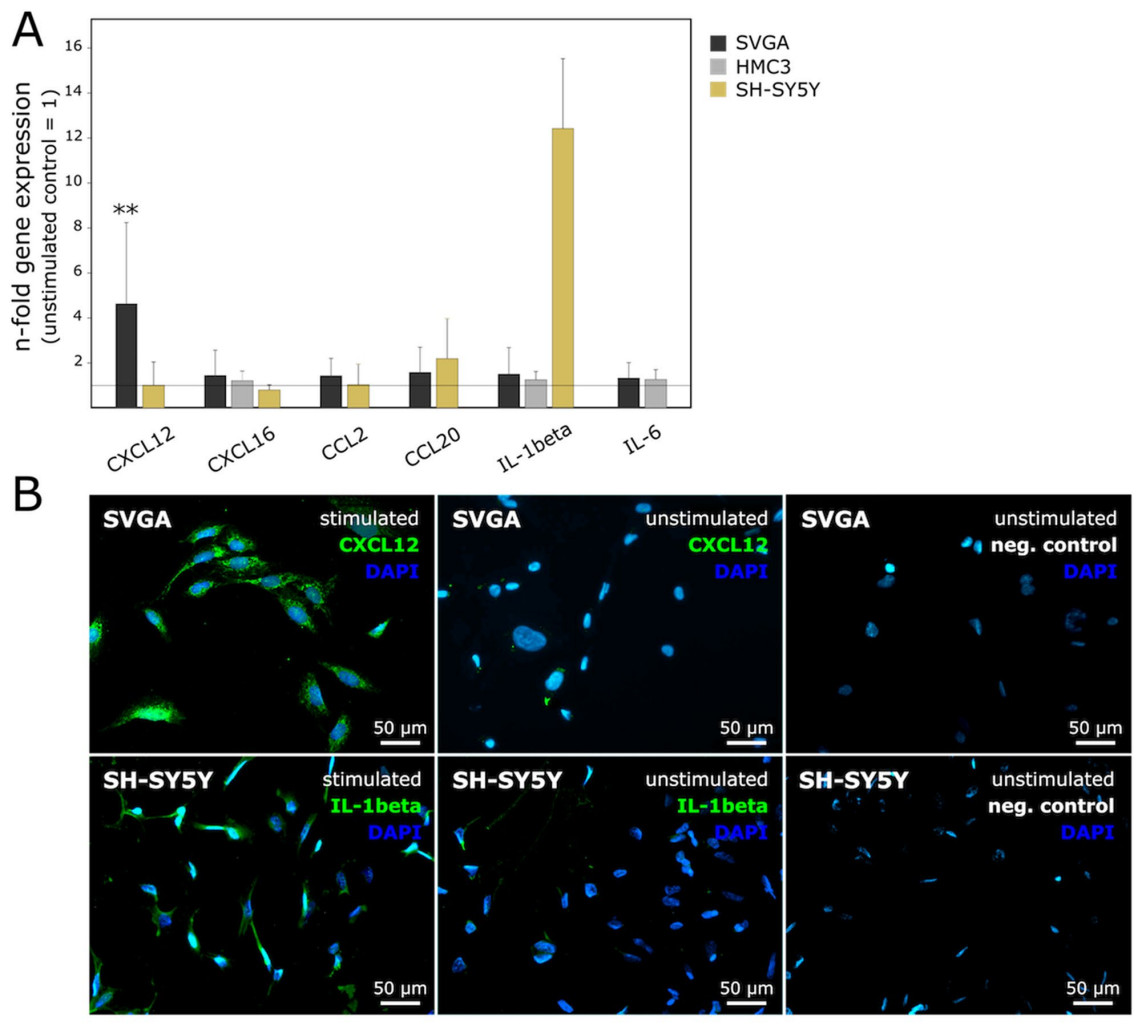
**A** N-fold gene expression differences of the inflammatory mediators (CXCL12, CXCL16, CCL2, CCL20, IL-1β, IL-6) between electrically stimulated and unstimulated SVGA (*n* = 8), HMC3 (*n* = 9) and SH-SY5Y (*n* = 9) cells calculated as $2^{{ - \left( {\Delta {\text{CT}}\,{\text{stimulated}}\, - \,\Delta {\text{CT}}\,{\text{unstimulated}}} \right)}}$. There was a statistically significant upregulation of CXCL12 (*p*: 0.034) in SVGA cells as determined by paired two-tailed Student’s *t* test of the linearized ΔC_T_ values, and a clear, but not statistically significant upregulation of IL-1β in SH-SY5Y. A solely qualitative evaluation of the immunofluorescence staining also showed a clearly stronger expression of the examined mediators at the protein level in the stimulated cells. Magnification ×200; bar = 50 μm

To evaluate the effects of electrical stimulation on the expression of the different chemokines and cytokines, the n-fold gene expression difference between stimulated and unstimulated cells was calculated for each cell line. Since CXCL12, CCL2, and CCL20 were not detectable in HMC3 and IL-6 not in SH-SY5Y cells, these mediators were not considered in the following examinations. A statistically significant upregulation of CXCL12 (*p*: 0.006; average ΔC_T stimulated_: 10.09, average ΔC_T unstimulated_: 11.85) in SVGA cells, and a clear, but not statistically significant upregulation of IL-1β (average Δ*C*_T stimulated_: 11.36, average ΔC_T unstimulated_: 14.96) in SH-SY5Y cells was found after electrical stimulation while the other mediators were not essentially affected by stimulation (Fig. 6A). To further validate our findings, immunofluorescence staining was performed in order to detect protein expression differences between stimulated and unstimulated cells with regard to CXCL12 and IL-1β. A solely qualitative assessment of the samples was carried out. The representative examples of fluorescence staining in Fig. 6B clearly illustrate the upregulation at the protein level of CXCL12 in SVGA and IL-1β in SH-SY5Y cells.

In summary, growth and survival of the examined human cell lines were not affected by electrical stimulation. This indicates that our in-vitro simulated DBS experimental setup could be used to study the influence of electrical stimulation on representative mediators involved in inflammation. After determining the basal expression of these inflammatory chemokines and cytokines in the studied human brain cell lines, electrical stimulation was found to upregulate the expression of some mediators at the mRNA as well as at the protein level. This indicates that electrical stimulation simulating the stimulation conditions in clinical use can influence inflammatory processes in various human brain cells.

## Discussion

DBS is a standard therapy in patients suffering from movement disorders such as PD, essential tremor or dystonia, and further indications have been proposed (Deuschl & Agid, 2013; Fenoy et al., 2018; Frizon et al., 2020; Huss et al., 2015; Huys et al., 2019; Janssen et al., 2014). Despite the effect on neuronal circuits leading to an improvement of symptoms, there are suggestions of an influence on proinflammatory cytokines and chemokines, e.g., reports of late onset edema under DBS. In most of these patients, the edema was not caused by an infection and resolved spontaneously within several months. The edema was probably caused by the inflammatory response to the irritation of implanting the DBS electrodes. Nevertheless, an additional effect of the stimulation is discussed, as this edema usually occurs several days after the operation, when stimulation has usually already been switched on (Cuba et al., 2016).

In order to investigate the influence of DBS on inflammatory processes in the central nervous system (CNS), we developed a new in-vitro model. As opposed to earlier studies which often used animal models and animal cell lines, respectively, we employed human cell lines in order to better transfer the results to patients. Furthermore, the applied stimulation was modeled to simulate the clinical settings. We stimulated with short impulses at a high frequency using the voltages that are applied to patients in the clinical routine. By using an indirect stimulation in our experimental setup, we avoided undesirable anion depositions in the medium, which could have otherwise falsified our results. In addition, the damage caused by implanting an electrode directly into the cell culture was avoided. Furthermore, the cells were placed on coverslips in order to avoid an uncontrolled spread of current. We were able to demonstrate that electrical stimulation itself did not influence cell proliferation or cell death. In this connection, cell counts and photomicrography did not reveal any differences in the growth behavior between stimulated and unstimulated cells. TUNEL assays of the stimulated and unstimulated cells only showed rare signs of apoptosis in either group.

While other studies using a continuous current showed a change in the morphology of the stimulated cells, we observed no morphological differences between stimulated and unstimulated cells in our study (Li et al., 2015; Pelletier et al., 2014).

Taken together, our model allows a valid investigation of the influence of simulated DBS on different cells of the CNS in vitro.

In a next step, we investigated the influence of electrical stimulation on proinflammatory cytokines and chemokines. Although CXCL16, CCL2, CCL20, and IL-6 were expressed in the most of the examined cell lines, we found no effect of the stimulation on the regulation of their gene expression. On the other hand, we found that the expression of CXCL12 was induced in the human astrocyte cell line SVGA, and that of IL-1ß was induced in human differentiated SH-SY5Y cells (dopaminergic-like neurons) at the mRNA level. The induction of the inflammatory mediators at the protein level was confirmed by immunocytochemistry. Our results are in accordance with those of Calleja-Castillo et al. who found an upregulation of IL-1ß after DBS of the hypothalamic nucleus in rats and, hence, suggested that immune responses might be altered in patients who are being treated with DBS (Calleja-Castillo et al., 2013).

CXCL12 attracts activated CXCR4 + T cells to the areas of inflammation (Nanki & Lipsky, 2000), and not least because of this important function, CXCL12, CXCR4, and CXCR7 receptors are attracting increased interest as therapeutic targets in a number of diseases (Ehtesham et al., 2013; Nanki & Lipsky, 2000)_ENREF_25. On the surface of differentiated neurons, particularly cholinergic and dopaminergic ones, CXCR4 represents the main target for CXCL12, but an interaction with CXCR7 at the intracellular level is also under investigation (Banisadr et al., 2002; Shimizu et al., 2011). Regarding neuroinflammation, CXCL12 and its receptors have been shown to be involved in the activation of microglia in a mouse model of PD. Furthermore, a positive correlation was found between α-synuclein, a protein involved in the regulation of dopamine release and associated with neurodegenerative disease, and CXCL12 in the postmortem brain tissue of PD patients (Li et al., 2019; Nanki & Lipsky, 2000). In a rat stroke model, Ruscher et al. demonstrated improved functional recovery due to inhibition of CXCL12 activity by housing the animals in an enriched environment that induced strong multisensory brain stimulation (Ruscher et al., 2013). Furthermore, there is evidence suggesting that CXCL12 plays a role in inflammation in multiple sclerosis (MS). In this connection, it has been found particularly on the walls of blood vessels indicating its involvement in leucocyte extravasation, and its pathological local expression has been shown to be associated with MS disease severity (Krumbholz et al., 2006; McCandless et al., 2008). McCandless et al. found opposing results when they inhibited CXCR4 in the setting of experimental autoimmune encephalitis: this resulted in the enhanced migration of infiltrating leukocytes into the white matter (McCandless et al., 1950). Although the involvement of CXCL12 in neuroinflammation is not yet fully understood, there are many indications that CXCL12 plays an essential role in the activation of inflammation.

The cytokine IL-1ß, which was, even though not to a statistically significant extent, induced by electrical stimulation in our experimental setup, was also shown to be involved in neuroinflammation. In MS, particularly, high levels of IL-1ß were found in the CSF, and its local concentration was furthermore associated with an increased cortical lesion load (Mendiola & Cardona, 1996). The excessive secretion of IL-1β by microglia was shown to cause neuronal death in rat models of stroke and PD (Mao et al., 2017; Yang et al., 2014). In Alzheimer`s disease, high levels of IL-1β were detected in microglial cells surrounding Aβ plaques and in the CSF of patients suffering from the disease, suggesting that this cytokine promoted neurodegeneration (Heneka et al., 2015). Nevertheless, there are also indications that IL-1ß induces the clearance of amyloid plaques by activating microglia cells (Mendiola & Cardona, 1996). An upregulation of IL-1ß by electrical stimulation, as observed in our study, was also shown by Calleja-Castillo et al. (Calleja-Castillo et al., 2013). Differing from our study, they administered 30 cycles of DBS, alternating 30 s of electrical stimulation with 30 s of rest. This was applied to the hypothalamic nucleus for 21 days in a rodent model (Calleja-Castillo et al., 2013). In addition to IL-1ß, increased serum levels of the proinflammatory mediators TNF-α, IL-6, and interferon-γ were detected by ELISA (Calleja-Castillo et al., 2013). Furthermore, the serum level of corticosterone was decreased under DBS. Since corticosterone suppresses inflammation, its low serum level might explain the observed upregulation of the proinflammatory cytokines. In accordance with the previously mentioned findings, Novakova et al. and Seifried et al. reported significantly decreased cortisol levels in PD patients after DBS of the subthalamic nucleus (Novakova et al., 2011; Seifried et al., 2013). De Koning et al. found similar results with decreased median levels of free cortisol in the urine of patients with obsessive–compulsive disorder who were receiving DBS of the nucleus accumbens (Koning et al., 2013).

Nevertheless, there is also evidence that DBS suppresses inflammation. For instance, Dandekar et al. detected a significant downregulation of IL-5 and IL-18 in the hippocampus and of IL-6 in the nucleus accumbens after electrical stimulation. To note, the study focused on the investigation of depression in a rat model, in which high levels of proinflammatory cytokines are usually present. Furthermore, elevated levels of TNF-α in the nucleus accumbens were significantly reduced by DBS. In this work, the effect of DBS was not only locally restricted, but BDNF levels in plasma and CSF were clearly increased by seven days of electrical stimulation, indicating a neuroprotective role of DBS (Dandekar et al., 2019).

Our results show a modulating effect of simulated DBS on cytokines and chemokines connected with inflammation, where CXCL12 and IL-1ß were induced by the stimulation. Since both cytokines have been shown to trigger neuroinflammation, their upregulation might explain the occurrence of late onset edema observed several days after DBS electrode implantation. To date, the effects of DBS on inflammation are still not fully understood. In addition, the clinical impact of these side effects caused by DBS, i.e., whether detrimental or neuroprotective remains a topic of discussion. Hence, further studies of the effects of elevated levels of CXCL12 and IL-1ß on relevant cells of the human central nervous system undergoing DBS are necessary.

## Conclusion

Using electrical stimulation simulating the stimulation used clinically in DBS, we were able to establish a valid in-vitro model for studying the influence of DBS on inflammatory mediators. In this framework, CXCL12 and IL-1ß were upregulated by electrical stimulation, revealing a clear effect of DBS on neuroinflammation. Further research is required to evaluate the clinical impact of the upregulation observed in our study.

## Future Work

In order to resemble the human in-vivo conditions as much as possible, novel 3D cultures might be a suitable advancement of our model to investigate the effects of electrical stimulation on inflammation in the different cells of the central nervous system in future. As an improvement of conventional human ex-vivo tissue and in-vivo animal models, organoids have been developed, which even enable the presence of complex vascular-like network (Cakir et al., 2019). To date, only a few studies use these models, e.g., in epilepsy or Alzheimer’s disease and studies focusing on electrical stimulation-induced inflammatory response are rare (Antill-O’Brien et al. 2019; Choi et al., 2014). In this context and in accordance with previous studies, Latchoumane et al. observed a significant upregulation of the expression of BDNF following direct current stimulation using embryonic stem cell-derived neuron and glial co-cultures (Latchoumane et al. 2018).

The aim of future research in the field of electrical stimulation-induced inflammatory response should be to further improve experimental setups, in order to closely mimic human conditions. Using suitable stimulation parameters and models, which resemble neuronal tissue forms and functions, molecular signaling, and the natural extracellular microenvironment might contribute to generate a better understanding of the complex phenomenon of inflammation caused by electrical stimulation.

## Data Availability

All data generated or analyzed during this study are included in this published article [and its supplementary information files].

## Abbreviations

BDNF: Brain-derived neurotrophic factor
BSA: Bovine serum albumin
cDNA: Complementary deoxyribonucleic acid
CCL2, CCL20: C-c motif chemokine ligand 2, 20
C_T_: Cycle of threshold
CXCL12, CXCL16: C-x-c motif chemokine ligand 12, 16
DBS: Deep brain stimulation
DF: Dilution factor
DMEN: Dulbecco’s modified Eagle’s medium
DSB: DNA strand breaks
EDTA: Disodium ethylenediaminetetraacetic acid
EMEN: Eagle’s minimum essential medium
FBS: Fetal bovine serum
GAPDH: Glycerinaldehyde 3-phosphate dehydrogenase
IgG: Immunoglobulin G
IL-1β and -6: Interleukine-1β, 6
INF: Interferon
ISIS: “Inomed” system for intraoperative stimulation
MS: Multiple sclerosis
NF200: Neurofilament 200
PBS: Phosphate buffered saline
PD: Parkinson’s disease
qRT-PCR: Real-time quantitative reverse transcription polymerase chain reaction
SD: Standard deviation
TdT: Terminal deoxynucleotidyl transferase
TNF-α: Tumor necrosis factor-α
TUNEL: Terminal deoxynucleotidyl transferase d-UTP nick end labeling
